# Extract of a Communication from Mr. Atkinson, in Answer to Dr. Kinglake

**Published:** 1816-12

**Authors:** 


*For the London Medical and Physical Journal.*

*Extract of a Communication from* Mr. ATKINSON, *in Answer to Dr. Kinglake.*

AT the commencement of this debate, Dr. K. complimented me upon the moderation and candour of my sentiments, in comparison with those of my coadjutors. I am sorry I should have forfeited his esteem: it was "the frankness of undisguised honesty," however, and not a wish to irritate the feelings of any man, which induced me to make use of some strong language, in what I conceive to be the cause of truth.

---

[The rest of Mr. A.'s paper contains nothing that we should not be proud to insert, had not so many of our readers expressed a fear that the controversy would become tedious, and for other reasons mentioned in a former Number.—EDIT.]

